# Genetically coating oncolytic herpes simplex virus with CD47 allows efficient systemic delivery and prolongs virus persistence at tumor site

**DOI:** 10.18632/oncotarget.26167

**Published:** 2018-10-02

**Authors:** Xinping Fu, Lihua Tao, Xiaoliu Zhang

**Affiliations:** ^1^ Department of Biology and Biochemistry and Center for Nuclear Receptors and Cell Signaling, University of Houston, Houston, Texas, USA

**Keywords:** oncolytic, herpes simplex virus, CD47, systemic delivery, innate antiviral

## Abstract

Current oncolytic virotherapy is primarily administered by intratumoral injection. However, systemic delivery is desirable for treating patients, particularly for those who have developed metastatic diseases. Several components are impeding the systemic delivery efficiency of oncolytic viruses. Chief among them is the rapid clearance of viral particles by the host’s mononuclear phagocyte system (MPS). We explored the possibility of genetically engrafting CD47, a “don’t eat me” signal molecule, to the membrane envelop of an oncolytic herpes simplex virus (HSV) to enable it to escape from the MPS for systemic delivery. Our results show that this modification indeed allows the virus to be more efficiently delivered to local tumors by the systemic route. Moreover, this modification also prolongs the virus persistence in local tumors after it arrives there. Consequently, systemic delivery of the modified virus produced a measurable antitumor effect against a murine tumor model that is otherwise resistant to the parental virus delivered by the same route. Our data thus suggest that engrafting enveloped oncolytic viruses such as those derived from HSV with CD47 molecule represents a conceivable strategy to enhance the efficiency of systemic delivery.

## INTRODUCTION

Herpes simplex virus (HSV) is the first virus that has been genetically modified for oncolytic purposes [1]. Twenty-five years later, a HSV-based oncolytic virus, T-VEC (Imlygic), is the first virotherapy to have been approved by the US Food and Drug Administration for clinical use [2]. Despite the exciting development, there are plenty rooms for improvement of virotherapy. For example, T-VEC and many other oncolytic viruses that are currently in various stages of clinical trials are primarily administered to cancer patients through intratumoral injection. However, systemic delivery is desirable for treating patients with many tumor types, particularly for those who have developed metastatic diseases. Despite the importance of this delivery route, an optimal systemic delivery strategy has not yet been developed. In our own experience, although therapeutic effect was detected in some experiments after oncolytic HSVs were delivered by the systemic route, the overall therapeutic efficacy was significantly less than those seen with intratumoral delivery [3, 4]. Several key components are impeding the delivery efficiency of oncolytic viruses by the systemic route. First, after systemic delivery, the viral particles are immersed in the large volume of blood and thus are immensely diluted. Second, a substantial portion of the infused viruses can be cleared rapidly by the host’s mononuclear phagocyte system (MPS) before they have any chance of reaching tumor tissues [5–7]. Macrophages are a key component of MPS. Consequently, studies by Fulci *et al.* have shown that depletion of macrophages can significantly improve the therapeutic effect of oncolytic HSV [8].

Like many cells from the host’s immune system, the activity of macrophages is controlled by both positive and negative regulatory mechanisms. Interactions between CD47 and its receptor, SIRPα, provide a strong negative regulation signal (“don’t eat me signal”) to macrophages [9]. An interesting recent study finds that coating nanoparticles with CD47 mimetic peptide can help them escape phagocytic clearance by MPS [10]. We explored the possibility of genetically engrafting the extracellular domain of CD47 molecule to the membrane envelop of an oncolytic HSV to enable it to escape from MPS for systemic delivery. Our results show that indeed this modification allows the virus to be more efficiently delivered to local tumors by the systemic route when compared to the parental virus. Moreover, this modification also makes the virus persist longer in local tumor after it arrives there. Our data thus suggest that engrafting an enveloped oncolytic virus such as the one derived from HSV with CD47 molecule represents a conceivable strategy to enhance systemic delivery as well as to prolong viral persistence in local tumors.

## RESULTS

### Virus modification strategy

**Figure 1:**
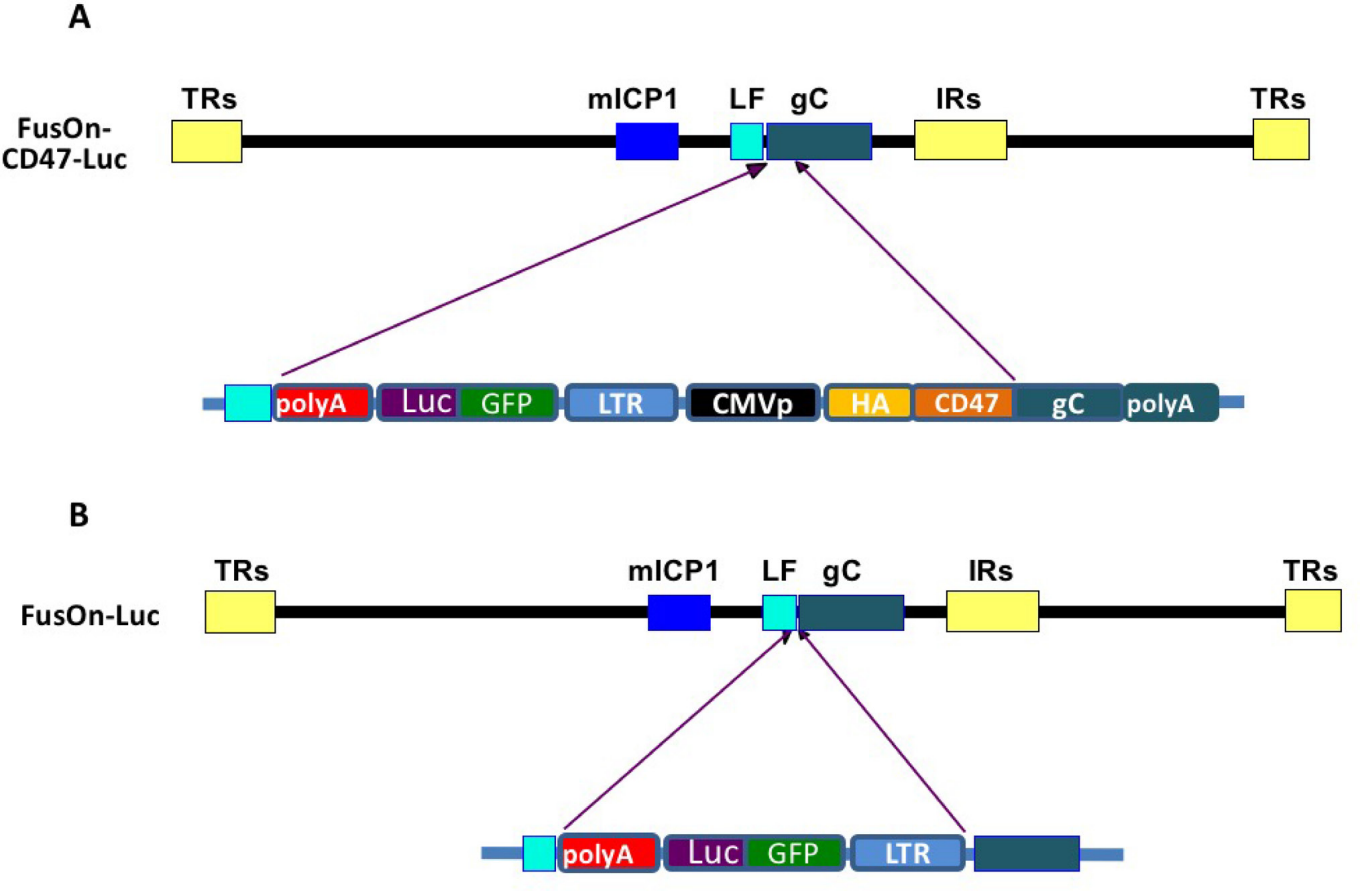
Schematic illustration of virus construction strategy (**A**) Insertion of EGFP-Luc gene cassette and the CMVp-HAtag-CD47ECD into the backbone of FusOn-H3. This was done through homologous recombination, with the left flanking (LF) sequence and the 3′ region of gC (from the transmembrane domain to the polyA) as the right flanking sequence. (**B**) Insertion of EGFP-Luc gene cassette alone into the 5′ side of the gC gene in FusOn-H3 as a control virus. The details of individual components in each gene cassette are depicted in lower part of the drawings and are labelled accordingly. The abbreviations are: CMVp, cytomegalovirus immediate early promoter; LTR, Rous Sarcoma long terminal repeats that contain the promoter region of this virus; GFP-Luc, EGFP-luciferase fusion gene; HA, HA tag; CD47, murine CD47 extracellular domain; gC, the complete gC coding region. LF, the left flanking region of gC. The recombinant viruses were identified by GFP expression and purified to homogeneity.

HSV encodes several glycoproteins that are assembled on the surface of viral envelope [11]. They include glycoprotein C (gC), gB, gD, gH and gL. Each of them can serve as a candidate molecule for incorporating the extracellular domain (ECD) of murine CD47 (mCD47) so that it may be engrafted to the surface of the virus envelope. We decided to choose gC, as unlike other glycoproteins mentioned, it is not essential for virus infectivity [12]. As such, modifying it for incorporating mCD47 would not run the risk of altering the natural tropism of the oncolytic virus. The details of virus construction strategy are illustrated in Figure 1. We initially inserted the ECD of mCD47 (aa 19–161) to the N-terminus of gC to create the chimeric form of gC (cgC), and its expression is driven by CMV IE promoter. We included a HA tag in cgC to allow the chimeric molecule to be conveniently detected. For the purpose of easiness in identifying the recombinant virus and for *in vivo* imaging, we linked cgC to another gene cassette that contains the EGFP-luciferase gene. These two gene cassettes were inserted together into the backbone of FusOn-H3, which was derived from a HSV-2 based oncolytic virus (FusOn-H2) by deleting the GFP gene from the virus. FusOn-H2 was constructed by deleting and replacing the N-terminal region of the ICP10 gene with *GFP* to render it the ability to selectively replicate in and kill tumor cells [13]. The recombinant virus was identified by picking up GFP positive plaques. Each individually picked virus was enriched to homogenous GFP positivity through multiple rounds of plaque purification. The newly generated virus is designated FusOn-CD47-Luc (Figure 1A). We also constructed a control virus in which only the EGFP-Luc gene cassette alone was inserted into the backbone of FusOn-H3, FusOn-Luc (Figure 1B). All the selected viruses maintain the fusogenic property of the parental FusOn-H2 [13].

### *In vitro* characterization of the newly constructed viruses

**Figure 2:**
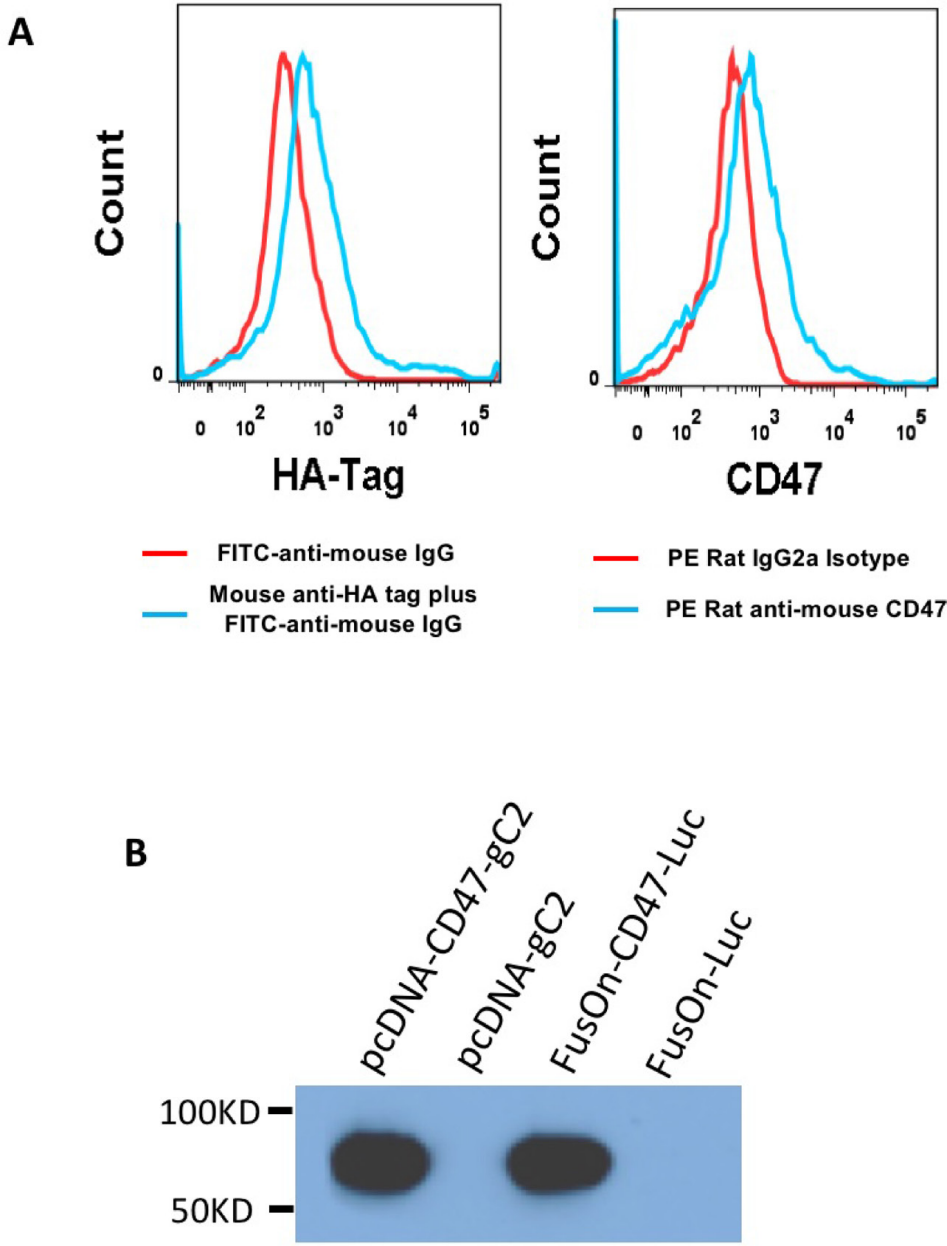
Assessment of transgene expression (**A**) Detection of cgC expression by flow cytometry. 293 cells were infected with either FusOn-CD47-Luc (blue line) or FusOn-Luc (red line) for 24 h. Cells were then stained with either mouse anti-HA-tag or PE conjugated anti-mCD47 IgG. A FITC-conjugated goat anti-mouse IgG was used as the second antibody for anti-HA tag staining. (**B**) Detection of cgC expression by Western blot. Cell lysates were prepared from 293 cells either infected with the viruses (FusOn-CD47-Luc or FusOn-Luc) or transfected with plasmids (pcDNA-CD47-gC2 or pcDNA-gC2). Western blot analysis was done on the cell lysates by using anti-HA tag IgG as the first antibody and horseradish peroxide conjugated goat anti-mouse IgG as the second antibody.

We initially examined the expression of the transgene by either flow cytometry or western blot analysis. For flow cytometry analysis, 293 cells were infected with 1 pfu/cell of either FusOn-CD47-Luc or FusOn-Luc. Twenty-four h later, cells were labeled with either a PE conjugated anti-mCD47 antibody or a rabbit anti-HA tag antibody. A goat-anti-rabbit antibody conjugated with FITC was added for HA tag detection. Both CD47 and HA tag labelled cells were subjected to flow cytometry analysis. The results in Figure 2A show that both anti-mCD47 and anti-HA tag antibodies were able to readily detect the cells that had been infected with FusOn-CD47-Luc, but not cells infected with FusOn-Luc. For western blot analysis, 293 cells were either similarly infected with these two viruses or transfected with plasmids that express cgC or wild type gC that does not contain the HA tag. Cell lysates were prepared 24 h later and were subjected to western blot analysis. The result in Figure 2B shows that cgC is abundantly expressed by FusOn-CD47-Luc but not FusOn-Luc.

**Figure 3:**
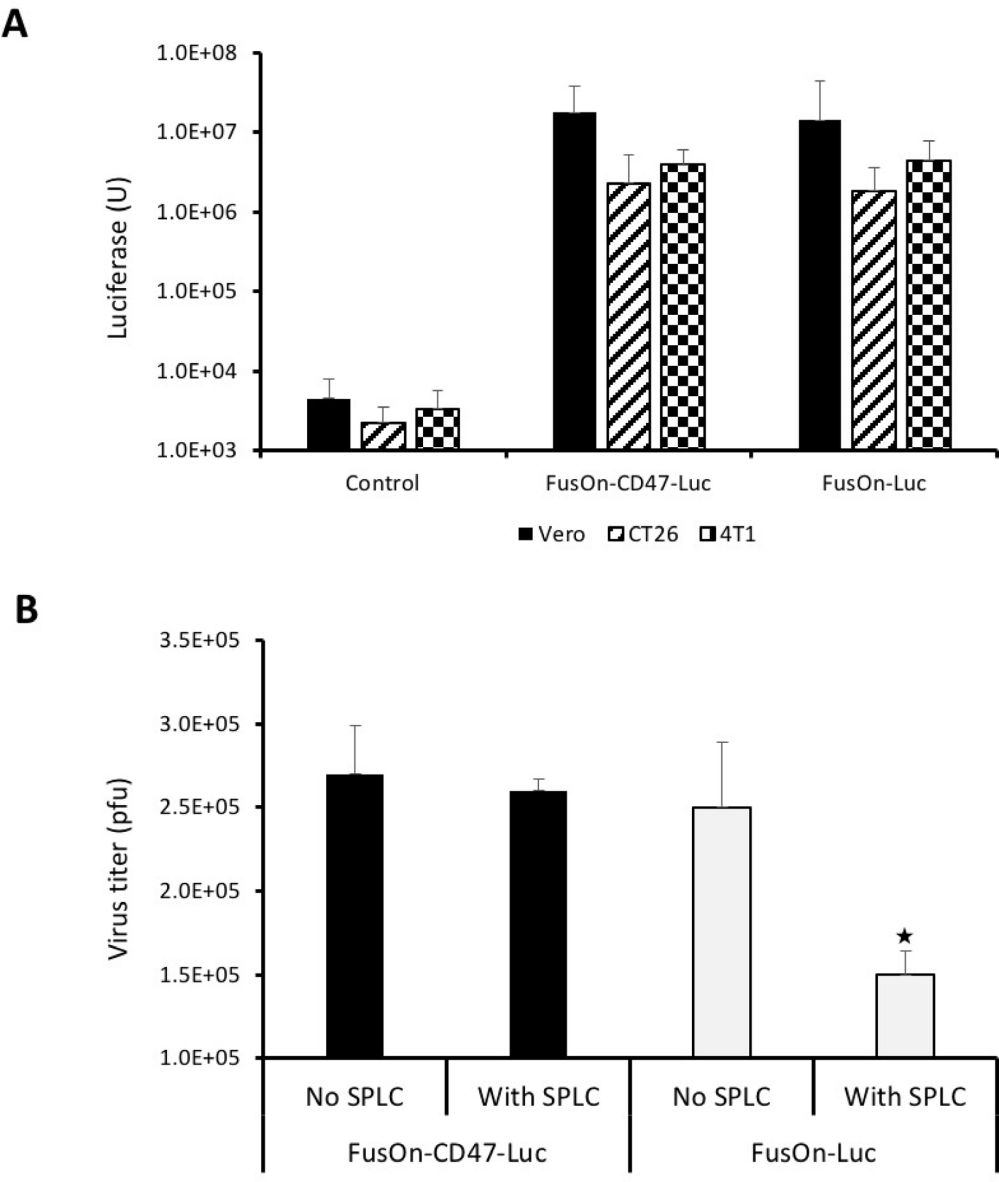
Comparison of FusOn-CD47-Luc and FusOn-Luc for luciferase expression and ability to evade clearance by phagocytes (**A**) Comparison of luciferase expression between these two viruses. Vero, CT26 and 4T1 cells were infected with the viruses at 1 pfu/cell and were harvested 24 h later for quantitation of luciferase activity. (**B**) Comparison of virus yield with or without the presence of phagocytes. Vero cells in 12-well plates were infected with either virus at 0.1 pfu/cell without or with the presence of 200,000 splenocytes. Cells were harvested 48 h and the virus yield was determined by plaque assay. ^★^*p* < 0.05 as compared with other three wells. The experiments with tumor cells (CT26 and 4T1) and Vero cells in A were conducted at different dates.

Next, we compared the quantify of Luc gene expression from these two viruses. We infected Vero, CT26 and 4T1 cells with either FusOn-CD47-Luc or FusOn-Luc at 1 pfu/cell. Cells were harvested 24 h later for measurement of luciferase activity. The result in Figure 3A showed a near identical level of luciferase activity from cells infected with these two viruses. We then conducted another experiment to determine, in the *in vitro* setting, if engrafting an oncolytic HSV with the ECD of mCD47 allows the virus to evade the engulfment and clearance by phagocytes. We used mouse splenocytes as the source of fresh phagocytes as spleen is the largest unit of the mononuclear phagocyte system [14]. We infected Vero cells with FusOn-CD47-Luc or FusOn-Luc with or without the presence of mouse splenocytes. Cells were collected 48 h later for quantitative measurement of virus yield by plaque assay. The results in Figure 3B showed that, in the wells without splenocytes, both FusOn-CD47-Luc and FusOn-Luc replicated well and the virus yield was similar in these two wells. However, in the wells with splenocytes, FusOn-Luc yield was significantly reduced while the replication of FusOn-CD47-Luc was only marginally affected. These results indicate that the presence of mCD47 ECD on viral particles enable the virus with the ability to resist the impact from macrophages and possibly some other immune cells during virus infection.

### FusOn-CD47-Luc can be effectively delivered to local tumors after systemic administration

**Figure 4:**
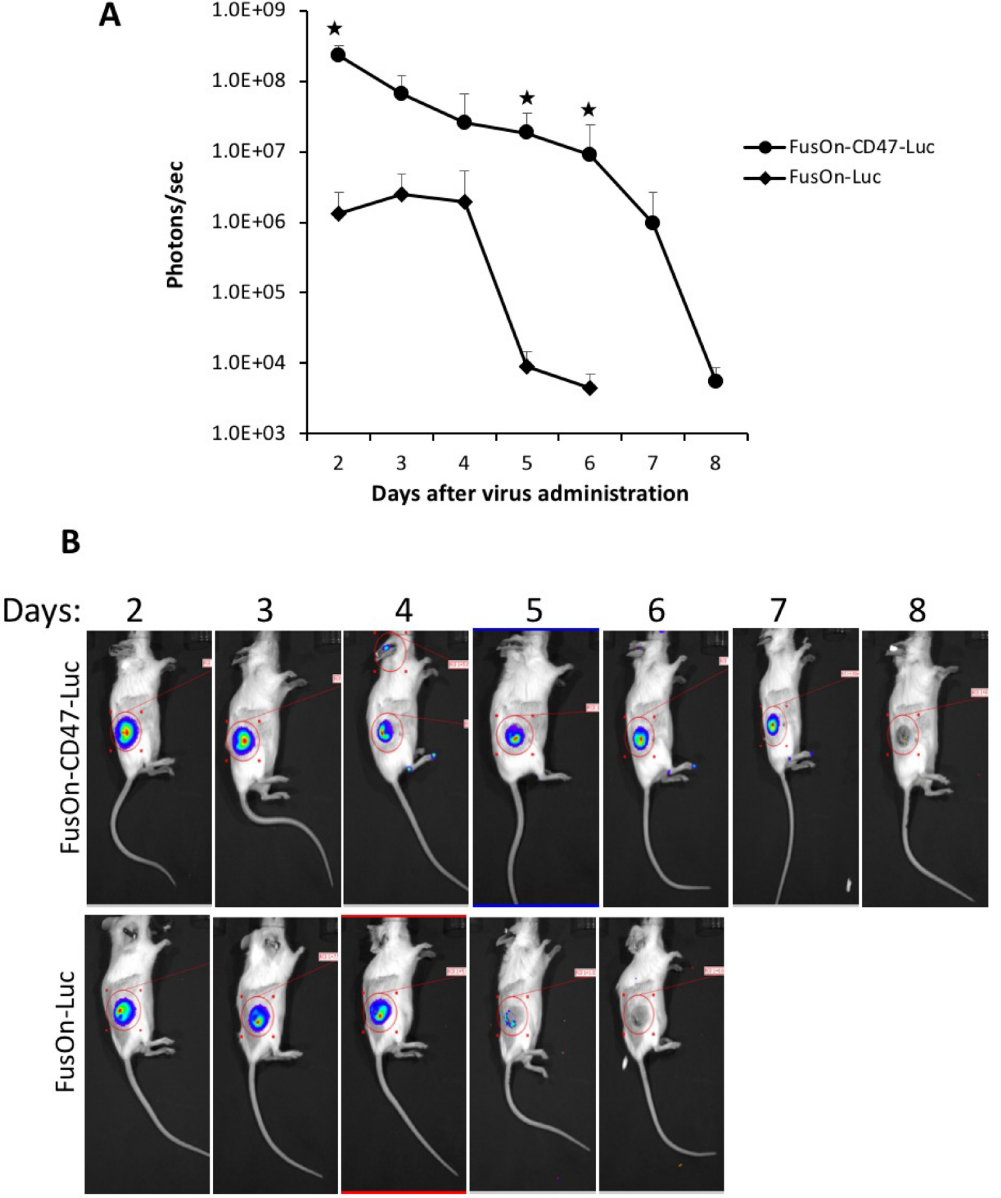
Comparison of FusOn-CD47-Luc and FusOn-Luc delivery by the systemic route in CT26 tumor model Tumor was established at the right flank of Balb/c mice by subcutaneous implantation of CT26 cells. Once tumor reached the approximate size of 8 mm in diameter, 2 × 10^6^ pfu of either FusOn-CD47-Luc or FusOn-Luc was given systemically. Animals were imaged daily starting on day 2 for luciferase expression. (**A**) Mean photon reading from daily IVIS imaging measurement. ^★^*p* < 0.01 as compared to FusOn-Luc. (**B**) A typical mouse from each group showing the actual imaging.

To determine the ability of the incorporated mCD47 ECD in enabling the virus for systemic delivery, we implanted CD26 murine colon cancer cells to the right flank of immunocompetent Balb/c mice. Once tumors reached the approximate size of 8 mm in diameter, we systemically injected 2 × 10^6^ pfu of either FusOn-CD47-Luc or FusOn-Luc to each mouse. Mice were monitored for luciferase activity by IVIS Spectrum System starting on day 2 and then on a daily basis until total disappearance of the signal. The results in Figure 4 show that, at day 2 after virus administration, the imaging signal from FusOn-CD47-Luc was approximately one and a half log stronger than that from FusOn-Luc. This indicates that the former was more efficiently delivered to the tumor site than later by the systemic route. Moreover, FusOn-CD47-Luc seems to stay in the tumor tissues substantially longer than FusOn-Luc. By day 5, the imaging signal was barely detectable in tumors from mice receiving FusOn-Luc, while the signal in tumors from FusOn-CD47-Luc remained detectable until day 7. Nevertheless, neither virus showed any significant amplification in tumor tissues because this HSV-2 based oncolytic virus grows poorly in CT26 tumor cells. Interestingly, in one occasion when a few mice were imaged at day 1 after virus delivery, significant imaging signals were detected transiently in the liver (Supplementary Figure 1). These signals completely disappeared by day 2.

**Figure 5:**
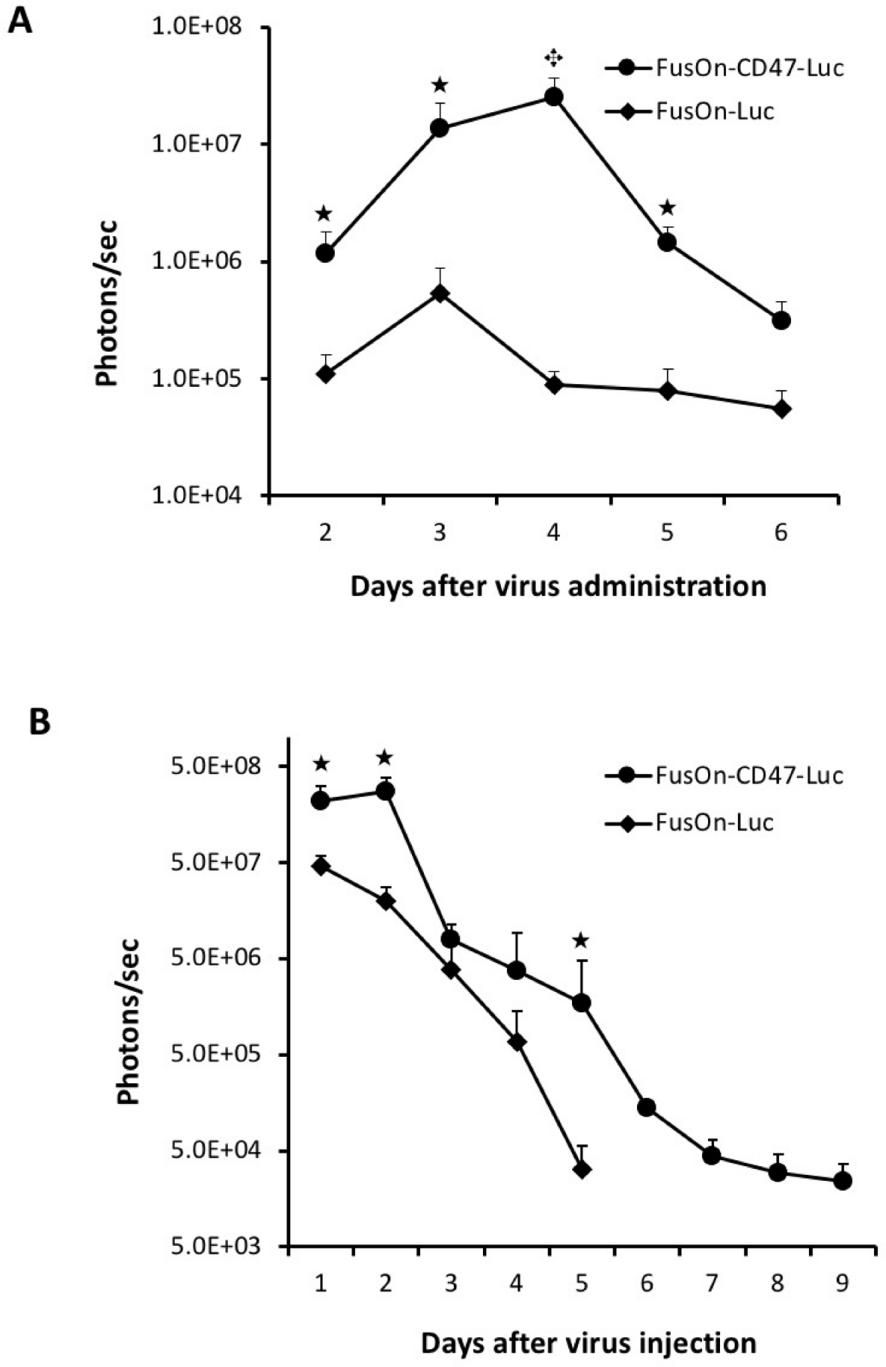
Comparison of FusOn-CD47-Luc and FusOn-Luc systemically delivered to 4T1 tumor and locally delivered to CT26 tumor (**A**) Tumor was established at the right flank of Balb/c mice by subcutaneous implantation of 4T1 cells. Once tumor reached the approximate size of 8 mm in diameter, 2 × 10^6^ pfu of either FusOn-CD47-Luc or FusOn-Luc was given systemically. Animals were imaged daily starting on day 2 for luciferase expression. ^★^*p* < 0.05, ^★^*p* < 0.01, as compared to FusOn-Luc. (**B**) Tumor was established at the right flank of Balb/c mice by subcutaneous implantation of CT26 cells. Once tumor reached the approximate size of 8 mm in diameter, 2 × 10^6^ pfu of FusOn-CD47-Luc and FusOn-Luc was injected directly into the tumor and the viral persistence was monitored by IVIS imaging for luciferase expression. ^★^*p* < 0.05 as compared to FusOn-Luc.

To determine if FusOn-CD47-Luc is also more superior than FusOn-Luc for systemic delivery in other tumor models, we repeated the above experiment but in mice bearing tumors at the right flank established from implantation of 4T1 murine mammary tumor cells, in which FusOn-H2 was found to be able to grow moderately [15]. Additionally, 4T1 tumor cells secrete macrophage colony stimulating factor (M-CSF) and granulocyte colony stimulating factor (G-CSF), which can enhance macrophage infiltration and phagocytosis [16–18]. This would allow the CD47-mediated evading strategy to be more vigorously tested. The IVIS imaging results in Figure 5A indeed showed that the signal in 4T1 tumor was in general lower than those detected in CT26 tumor (as shown in Figure 4). Nevertheless, the results again showed that FusOn-CD47-Luc could be more efficiently delivered to local tumors than FusOn-Luc by the systemic route. By day 2, difference of the image signal strength between these two viruses was about one and half a log. The biggest difference was recorded on day 4, when the image signal from FusOn-Luc was reduced to nearly background level while it reached the highest for FusOn-CD47-Luc. These data again showed that CD47 modification allows the virus to be more efficiently delivered by the systemic route as well as persisted in tumor tissues longer than the control virus once it had reached there.

### Viral persistence after intratumoral delivery

The data in Figures 4 and 5A suggest, in addition to be more efficiently delivered to tumor site by the systemic route, FusOn-CD47-Luc also persisted in local tumors longer than FusOn-Luc. To examine this more directly, we compared for the virus persistence in tumor tissues after both viruses were given intratumorally to tumors established from subcutaneous inoculation of CT-26 tumor cells. As the virus was delivered intratumorally, we started to monitor luciferase activity by day 1 after virus injection and this was done again on a daily basis afterwards. The results in Figure 5B showed that FusOn-CD47-Luc indeed persisted longer than FusOn-Luc in the tumor tissues after direct local delivery. This result indicates the prolonged persistence of FusOn-CD47-Luc in tumor tissues is the direct result of mCD47 ECD, and not due to other explanations from the systemic delivery route.

### Evaluation of *in vivo* therapeutic effect after systemic delivery

**Figure 6:**
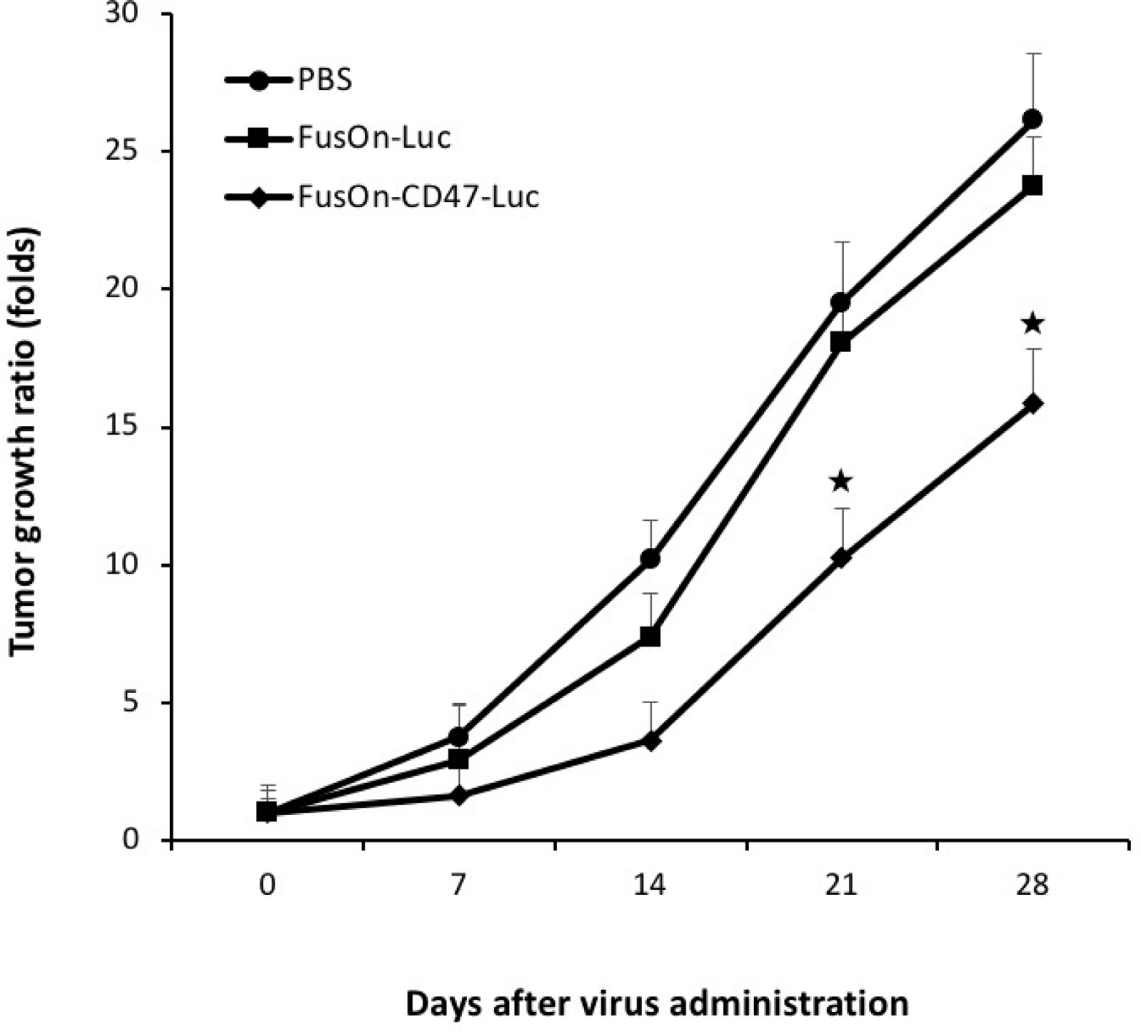
Therapeutic evaluation of virotherapy against CT26 tumor after systemic delivery of oncolytic viruses 2 × 10^6^ pfu of FusOn-CD47-Luc or FusOn-Luc was given to tumor-bearing mice systemically. Another group of mice was given PBS as a negative control. Tumor size was measured weekly and was presented as tumor growth ratio, which was determined by dividing the tumor volume measured on the indicated time after virus injection by the tumor volume before treatment. ^★^*p* < 0.05 as compared to FusOn-Luc.

In our previous work, oncolytic HSVs were found to show only a moderate therapeutic effect when they were given by the systemic route to treat fully permissive xenografted tumors in immunodeficient mice [3, 4]. To determine if mCD47 ECD engraftment would enable the virus to exhibit therapeutic efficacy against minimally permissive murine tumors in immunocompetent mice by the systemic delivery, we again established CT26 tumors subcutaneously in Balb/c mice. Once tumors reached the approximate size of 5 mm in diameter, we treated the animals with 2 × 10^6^ pfu of either FusOn-CD47-Luc or FusOn-Luc by the systemic route. Tumor size was measured weekly for 4 weeks the tumor volume data are plotted in Figure 6. FusOn-Luc, when delivered by the systemic route, did not show significant impact on the growth of this murine tumor formed from CT26 cells which are only minimally permissive to this HSV-2-based oncolytic virus. This result is consistent with what we repeatedly saw in our previous experiments (unpublished data). When delivered with the same dose and by the same route, FusOn-CD47-Luc showed a moderate but statistically significant impact on slowing down tumor growth. Together, these data show that the enhanced systemic delivery enabled by mCD47 ECD translates into improved therapeutic benefit.

## DISCUSSION

With the historical approval of T-VEC (Imlygic) by the FDA for treating patients with advanced melanoma, it is anticipated that more virotherapies may be approved for clinical use in the near future. T-VEC and other oncolytic viruses in various stages of clinical trials are all given by local injection. For many cancer patients, and particularly those with metastatic diseases, systemic administration will provide additional therapeutic benefits. However, there are many obstacles facing the delivery of oncolytic viruses by the systemic route. One of them is the rapid clearance of viral particles by the body’s mononuclear phagocyte system (MPS). The main components of MPS are monocytes/macrophages. They can rapidly clear viruses in the blood through phagocytosis [19], and thus represent a major obstacle for systemic delivery of oncolytic viruses. In order to overcome this obstacle, we investigated the possibility of genetically engrafting an oncolytic HSV with the CD47 molecule to allow it to escape phagocytosis by macrophages. This strategy is based on the finding that CD47 functions as a strong inhibitory signaling regulator on phagocytosis by binding to the SIRPα receptor on macrophages and other phagocytic leukocytes [20]. The binding of CD47 ECD with SIRPα results in tyrosine phosphorylation of its cytoplasmic immunoreceptor tyrosine-based inhibition motifs (ITIMs), leading to inhibitory signaling events and the consequential prohibition of phagocytosis [21]. Our data indeed show that engrafting the oncolytic HSV viral particles with the ECD of mCD47 allows the virus to escape clearance by macrophages when tested *in vitro* and enables the virus to be more efficiently delivered to tumor tissues by the systemic route when tested in two separate animal models. Although we conducted the experiments primarily on mice bearing local tumors, we expect that the CD47 approach would allow the oncolytic virus to be more efficiently delivered to metastatic tumors as well by the systemic route.

Previous studies by Fulci *et al.* showed that pretreatment with cyclophosphamide during oncolytic HSV virotherapy on brain tumors can significantly inhibit macrophage infiltration and this resulted in a significant enhancement of virus replication and hence the therapeutic efficacy [22]. Another study from the same group showed that direct depletion of peripheral macrophages and brain microglia with clodronate liposomes *in vivo* through systemic delivery and *ex vivo* in brain slice models with gliomas can dramatically increase the local oncolytic HSV titers [8]. Our own early studies also showed that co-administration of cyclophosphamide could significantly improve the therapeutic effect of FusOn-H2 against lung cancer [23]. Although these studies have drawn a consensus conclusion that antagonizing the phagocytic effect of macrophages on oncolytic HSVs during virotherapy is beneficial for the therapeutic outcome, translating these strategies into clinical application may be problematic. First, some of these depleting reagents (e.g., clodronate liposomes) may not be suitable for use in patients. Second, these strategies can result in systemic depletion of macrophages, which may lead to unforeseen side effects. Our strategy of engrafting oncolytic HSVs with the CD47 ECD through genetic conjugation to gC renders the virus with the intrinsic ability to evade the phagocytic clearance of macrophages without the need for adding additional chemical drugs to deplete them. This makes the clinical translation of the modified virus more feasible.

Our data also demonstrate that FusOn-CD47-Luc persisted longer than FusOn-Luc after the viruses had reached to the tumor site after systemic delivery or after being injected directly into the tumor tissue. This probably did not come out as a surprise, considering that resident macrophages play an important role in host’s defense against HSV and other virus infections [7]. We envisage that engrafting an oncolytic virus with the CD47 ECD allows the virus to initially escape the phagocytic clearance when the virus is administered systemically into the blood stream. It will then enable the virus to persist longer and replicate better after the virus has reached the tumor site, probably by preventing macrophages from clearing both the progeny viruses as well as the infected tumor cells that may have cgC expressed on the plasma membrane. Together, they seem to have contributed to an enhanced antitumor effect that we have shown in a murine tumor model that is otherwise only minimally permissive to the oncolytic effect of FusOn-H2, allowing the virotherapy to show a measurable antitumor effect after the virus is systemically administered.

## MATERIALS AND METHODS

### Cell lines and viruses

African green monkey kidney (Vero) cells, 293 cells and CT26 murine colon cancer cells were purchased from American Type Culture Collection (Manassas, VA, USA). 4T1 cells, a 6-thioguanine-resistant cell line derived from a BALB/c spontaneous mammary carcinoma [24], was kindly provided by Dr. Fred Miller (Michigan Cancer Foundation, Detroit, MI, USA). Cells were cultured with DMEM containing 10% fetal bovine serum (FBS). All media contained 100 U ml^–1^ penicillin and 100 mg ml^–1^ streptomycin (Invitrogen, Carlsbad, CA, USA). The details of the construction of the HSV-2-based FusOn-H2 have been reported [13, 25]. FusOn-H3 was derived from FusOn-H2 by removing the *GFP* gene from the viral genome through homologous recombination. The virus was identified by picking a white plaque from the green background and *GFP* deletion was confirmed by DNA sequencing of the impacted region of the viral genome.

### Construction of FusOn-CD47-Luc and FusOn-Luc

The detailed strategy of recombinant virus construction is illustrated in Figure 1. The gC left flank sequence and the entire gC sequence (also serves as the right flank) were amplified from HSV-2 186 strain with the following pairs of primers (in bold): primer pair for amplifying the left gC flanking sequence, forward 5′-TCGCGA CTGTTTGTCGGCACCCT GG and reverse 5′-ACGCGTTTAATTAACAGCGAGG AGGGTTGCC G; primer pair for amplifying the entire gC sequence, forward 5′-GCTAGCAAGCTTTGTAC AAATGCCTC CCCCGGACG and reverse 5′-CTCGA GCCGCAGCCGACGATAGC. DNA sequence of murine CD47 extracellular domain (GenBank: Z25524.1) corresponding to amino acids 19–161, plus a leader and a HA tag, was optimized and synthesized by GenScript (Piscataway, NJ, USA). The synthesized mCD47 ECD and PCR-amplified gC coding sequence were cloned into pcDNA-HA so that they were linked in frame, and the new plasmid is designated pcDNA-CD47-gC2. A control plasmid, pcDNA-gC2, is similarly constructed but does not contain mCD47 ECD and HA tag. Next, the PCR-amplified left flank sequence and a GFP-luciferase cassette driven by Rous Sarcoma LTR were inserted into pcDNA-CD47-gC2 or pcDNA-gC2 to the 5′ side of mCD47 ECD – HA tag-gC or gC, and the new plasmids were designated pcDNA-CD47-gC-gfpluc and pcDNA-gC-gfpluc, respectively. The DNA of these two plasmids were mixed with FusOn-H3 DNA at a 1:1 ratio in a total of 2 µg and then cotransfected into Vero cells using lipofectamine 2000 (ThermoFisher, Walthan, MA, USA). The recombinant viruses generated from homologous recombination were identified by picking up green plaques and subsequently confirmed by DNA sequencing of the viral genome region that contains the inserted sequence. The newly produced viruses were designated FusOn-CD47-Luc and FusOn-Luc, respectively.

### Flow cytometry detection of cgC expression from the modified virus

In order to confirm the chimeric gC (cgC) expression from the recombinant virus and that the expressed cgC is anchored on cell surface, 293 cells were infected with 1 pfu/cell of either FusOn-CD47-Luc or FusOn-Luc. Cells were harvested 24 h later and stained either with 5% PE anti-mouse CD47 antibody (BioLegend, San Diego, CA, USA) or first with 1% mouse anti-HA tag and then with 2% goat anti-mouse -FITC antibody (Sigma, St. Louis, MO, USA) in 2% FBS-PBS at 4° C for 30 min. Cells stained with either PE Rat IgG or FITC goat anti-mouse IgG as the isotype control. The stained cells in 2% FBS-PBS were analyzed by flow cytometry (BD Biosciences, San Jose, CA, USA).

### Western blot detection of cgC expression from the modified virus

293 cells were either transfected with pcDNA-CD47-gC2 or pcDNA-gC2, or infected with FusOn-CD47-Luc or FusOn-Luc at 1 pfu/cell. Cells were harvested 24 h later and treated on ice for 30 min with RIPA buffer (150 mM NaCl, 1.0% NP-40, 0.5% sodium deoxycholate, 0.1% sodium dodecyl sulphate, 50 mM Tris-HCl pH 8.0) that contains the protein inhibitor cocktail cComplete (Roche, Mannheim, Germany). This was followed by sonication one time for 15 seconds. The supernatants were collected after centrifugation at 18,000 × g for 20 minutes in a 4° C pre-cooled centrifuge. After adding 2 × Laemmli sample buffer (Bio-Rad, Hercules, California, USA), the samples were boiled for 5 min before they were loaded to a 12% SDS-PAGE gels (Bio-Rad) for electrophoresis. The separated proteins in the gel were then electroblotted onto PVDF membranes (Bio-Rad). All blots were blocked with 3% BSA in TBS-Tween (0.1%) at room temperature for 1 h, and then labeled with a primary antibody which is rabbit anti-HA tag (Cell Signaling, Danvers, MA, USA) at 1:1000 at 4° C overnight. A secondary antibody, goat anti-rabbit conjugated to horseradish peroxidase (Cell Signaling, Danvers, MA, USA), was diluted at 1:2000 and used for visualization by chemiluminescence.

### *In vitro* virus replication assay

To determine the impact of phagocytes on virus yield, Vero cells in 12-well plates were infected with either FusOn-CD47-Luc or FusOn-Luc at 0.1 pfu/cell without or with the presence of 200,000 splenocytes which were harvested from Balb/C mice. Cells were harvested 48 h later and the virus yield was determined by plaque assay on Vero cells.

### *In vitro* Luciferase reporter assay

Vero, CT26 and 4T1 cells were plated in 12-well plates one day before infection. Cells were infected with 1 pfu/cell of either FusOn-CD47-Luc or FusOn-Luc for 24 h before they were harvested. Luciferase activity was quantified with the Bright-Glo™ Luciferase Assay System (Promega, Madison, WI, USA) and readings were taken on the luminometer (GloMax^®^ 96 Microplate Luminometer, Promega) using FluoroNunc™/LumiNunc™ Plates (Fisher Scientific, Pittsburgh, PA, USA). Relative light units (RLU) were corrected based on total protein concentrations determined by the Bradford Protein Assay Kit (Bio-Rad, Hercules, CA, USA).

### Animal studies

Six-week old Balb/c mice were purchased from Jackson Laboratories (Indianapolis, IN, USA). All animal experimental procedures were approved by the University of Houston Animal Care and Use Committee. CT26 and 4T1 cells were cultured in DMEM in standard conditions until 70% confluence. Cells were then trypsinized, pelleted and resuspended in DMEM at a concentration of 2 × 10^6^ per ml. 2 × 10^5^ (in 100 µl) were subcutaneously implanted into the right flank of mice. Mice were then randomly divided into groups (*n* = 6 mice per group). For evaluating the therapeutic efficacy of virotherapy, mice received intravenous injection of 2 × 10^6^ pfu of oncolytic viruses (or PBS as a negative control) when tumor reached the approximate size of 5 mm in diameter. Tumor growth was monitored weekly by bidirectional measurements using a caliper, and the tumor volume was calculated by the formula [mm^3^] = (length × (width)^2^/0.5.

For evaluating viral delivery and persistence at the tumor site, mice received intravenous or intratumoral injection of 2 × 10^6^ pfu of either FusOn-CD47-Luc or FusOn-Luc when tumor at a size of 8 mm in diameter (*n* = 6). *In vivo* imaging was performed under an IVIS Spectrum Pre-clinical *in vivo* Imaging System (Perkin Elmer, Waltham, MA, USA) for the observation of luciferase activity. Briefly, mice were injected intraperitoneally with 150 mg/kg D-luciferin (Gold Bio technology, St. Louis, MO, USA) and then anesthetized with 1–3% isoflurane. Bioluminescence images were taken 10 min after the luciferin injection. Images were analyzed using Living Image version 4.2 software (Perkin Elmer) and represented as total flux measurements in photons/second.

### Statistical analysis

All quantitative data are reported as mean ± standard deviations (SD). Statistical analysis was made for multiple comparisons using analysis of variance and Student’s *t*-test. *p*-value < 0.05 was considered to be statistically significant.

## SUPPLEMENTARY MATERIALS FIGURE


