# Supplementary material for: Genetically coating oncolytic herpes simplex virus with CD47 allows efficient systemic delivery and prolongs virus persistence at tumor site
Source: Oncotarget. 2018 Oct 2;9(77):34543–53. doi: 10.18632/oncotarget.26167 (PMC6195384; doi:10.18632/oncotarget.26167)
Supplement: Supplementary file 1 [file oncotarget-09-34543-s001.pdf]

Genetically coating oncolytic herpes simplex virus with CD47 allows efficient systemic delivery and prolongs virus persistence at tumor site

SUPPLEMENTARY MATERIALS

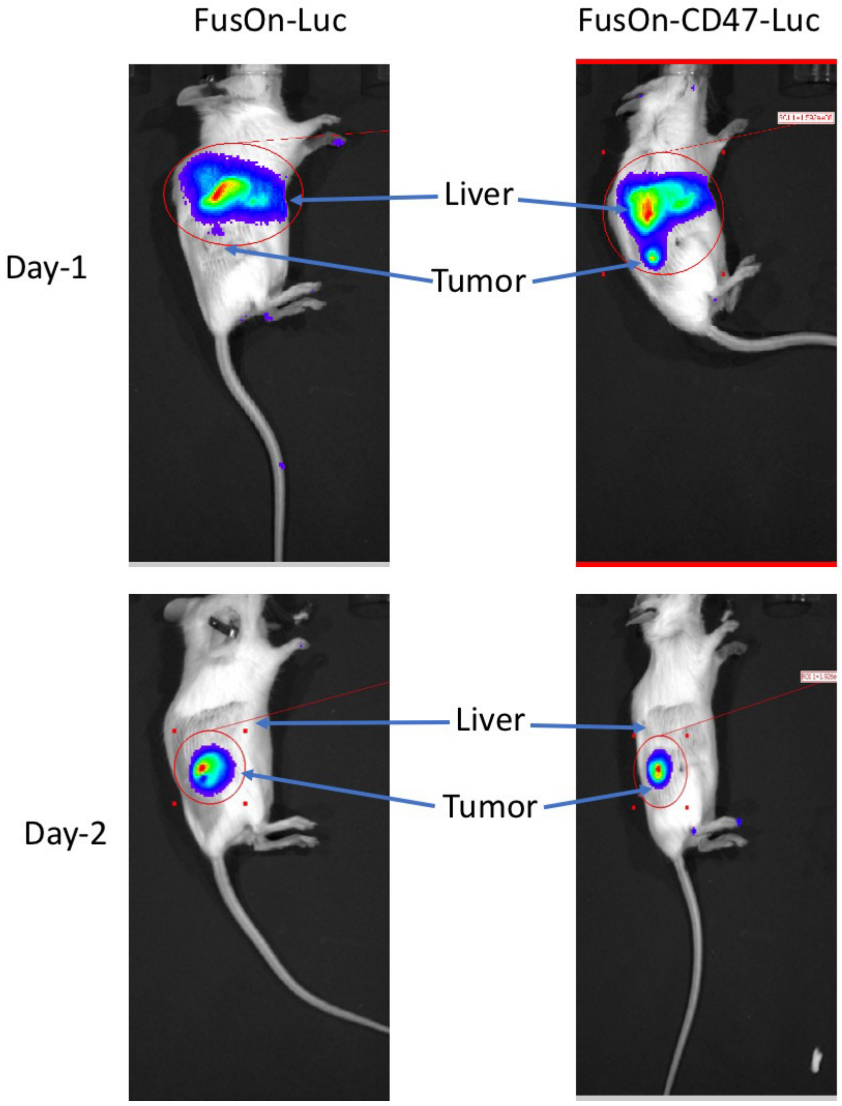

Supplementary Figure 1: Transient detection of both FusOn-CD47-Luc and FusOn-Luc in liver after systemic delivery. Tumor was established at the right flank of Balb/c mice by subcutaneous implantation of CT26 cells. Once tumor reached the approximate size of 8 mm in diameter, 2×10^6 pfu of either FusOn-CD47-Luc or FusOn-Luc was given systemically. Animals were imaged on day 1 and day 2 for luciferase expression. The location of IVIS image was indicated by arrows.
